# Comprehensive genomic profiling for advanced hepatocellular carcinoma in clinical practice

**DOI:** 10.1007/s12072-024-10741-y

**Published:** 2024-11-14

**Authors:** Takeshi Terashima, Tatsuya Yamashita, Kuniaki Arai, Noboru Takata, Tomoyuki Hayashi, Akihiro Seki, Hidetoshi Nakagawa, Kouki Nio, Noriho Iida, Shinya Yamada, Tetsuro Shimakami, Hajime Takatori, Kunihiro Tsuji, Hajime Sunagozaka, Eishiro Mizukoshi, Masao Honda, Shinji Takeuchi, Taro Yamashita

**Affiliations:** 1 Department of Gastroenterology, Kanazawa University Hospital, 13-1 Takaramachi, Kanazawa, Ishikawa 920-8641 Japan; 2 Department of Gastroenterology, Ishikawa Prefectural Central Hospital, Kanazawa, Japan; 3 Department of Gastroenterology, Fukui Prefectural Hospital, Fukui, Japan; 4 Division of Medical Oncology, Cancer Research Institute, Kanazawa University, Kanazawa, Japan

**Keywords:** Hepatocellular carcinoma, Comprehensive genomic profiling, Second-line treatment, Precision medicine, Gene alteration

## Abstract

**Aim:**

Although several therapeutic agents show efficacy in advanced hepatocellular carcinoma (HCC), biomarkers such as comprehensive genomic profiling (CGP) for the selection of second-line treatments after immunotherapy have not been established. We evaluated the value of CGP for the treatment decision in patients with HCC.

**Methods:**

We retrospectively studied 52 patients with advanced HCC who received CGP tests at three tertiary hospitals between February 2022 and November 2023. Genomic profiles were obtained using one of three CGP tests; 49 and 3 patients were evaluated using tissue-based and blood-based assay, respectively. The impact of CGP results on subsequent treatment selection in clinical practice and correlations between representative gene alterations and patient characteristics or responses to immunotherapy were evaluated.

**Results:**

The most frequently observed variants were *TERT* mutations*,* followed by *CTNNB1*, *TP53*, *ARID1A*, and *MYC* mutations. Potentially druggable gene alterations were observed in 45 patients (87%), and 34 patients (65%) were recommended to receive treatments based on specific gene alterations by a molecular tumor board. Treatments were covered by health insurance in 13 patients (25%). Five patients (10%) received the recommended treatment by the date of data cut-off. There were no differences in the efficacy of immunotherapy with respect to mutation status in *hTERT*, *CTNNB1*, *TP53*, *ARID1A*, and *MYC*.

**Conclusions:**

The results of the present study suggested that druggable gene alterations may provide useful information not only in proposing alternative treatment after standard of care but also in selecting second-line targeted treatments after immunotherapy for patients with advanced HCC.

## Introduction

Hepatocellular carcinoma (HCC) is the sixth most common cancer and the third leading cause of cancer-related mortality worldwide [1]. New imaging techniques for high-risk patients have improved early detection, and drugs that eradicate hepatitis viruses have reduced the occurrence of HCC [2, 3]. However, patients who undergo curative treatment frequently experience multicentric recurrence and intrahepatic metastasis. Recurrent HCC invades the vasculature or metastasizes to extrahepatic sites and becomes refractory to locoregional therapies.

Sorafenib and lenvatinib improved patient outcomes in a clinical trial for advanced HCC [4, 5]. Two immunotherapy regimens are beneficial as first-line therapies [6, 7], and these are now part of the standard of care. In addition, several agents, such as regorafenib, ramucirumab, and cabozantinib, are widely used in later-line therapy [8–10]. However, the most effective therapies after first-line immunotherapies have not been established.

Comprehensive genomic profiling (CGP) tests for patients with cancer can provide clinically significant information for understanding cancer development and progression, for the development of new therapeutic agents, and for the development of personalized medicine based on gene alterations in individual patients. These tests are widely used in daily clinical practice, especially for colorectal, pancreatic, and biliary tract cancers [11].

The aim of the present study was to characterize the genomic profiles of patients with advanced HCC and to investigate the impact of CGP results on subsequent treatment selection in clinical practice. Moreover, we explored the correlations between representative gene alterations and both patient characteristics and the effects of immunotherapy.

## Methods

### Patients

Patients with advanced HCC who received CGP tests at Kanazawa University Hospital, Ishikawa Prefectural Central Hospital, and Fukui Prefectural Hospital in Japan from February 2022 to November 2023 were included. HCC was basically diagnosed according to the radiological findings of hyperattenuation in the arterial phase and hypoattenuation in the late phase, which were revealed using dynamic computed tomography (CT) or magnetic resonance imaging (MRI) following the guidelines of the American Association for the Study of Liver Disease [12]. Patients included in this study received tumor biopsy for pathological confirmation or for future CGP examination and pathologically diagnosed as HCC. All patients also underwent CT scanning of the chest to pelvic area to assess the extent of HCC. Patients received systemic therapy with the optimal regimen recommended by each institutional cancer board, although atezolizumab and bevacizumab combination therapy was established as the standard of care during the study period. Patients underwent dynamic CT or dynamic MRI every 6–9 weeks to assess the efficacy of treatment. Anti-tumor effects were assessed in accordance with the Response Evaluation Criteria in Solid Tumors (RECIST) ver. 1.1.

### CGP tests

Tissue samples for CGP tests were obtained using a disposable biopsy instrument (18G needle), and a specimen of sufficient volume for CGP was obtained. In patients who had difficulty obtaining biopsy samples from HCC at advanced stage, previously resected specimens were used. Three CGP tests, OncoGuide™ NCC OncoPanel System (Kobe, Japan), FoundationOne® CDx (Cambridge, MA, USA), and Foundation One® CDx Liquid (Cambridge, MA, USA), are available in Japan. These three kinds of CGP testing were different with regard to the number of genes covered or the depth of coverage. All three platforms are approved for insurance-based clinical practice; however, insurance only covers one CGP test per patient in Japan. At our institution, we use FoundationOne® CDx preferentially owing to its detection sensitivity and number of targeted genes. When sample quantity is limited, we select the NCC OncoPanel System, and in the absence of tissue samples, we utilize FoundationOne Liquid CDx. We think that this approach ensures optimal testing based on the clinical context. CGP results were interpreted by members of a molecular tumor board [13] to determine recommended treatments based on gene alterations. All patients were provided comprehensive information about CGP tests, including the purpose, method, cost, and possibility of incidental findings, as well as information about safety and efficacy of treatments. All patients provided written informed consent.

### Data collection

We retrospectively collected demographic, clinical, and laboratory data, including comprehensive actionable and druggable gene alterations. Targeted treatments were recommended based on targeted gene alterations by the molecular tumor board. The Institutional Review Board of each hospital approved this study, which was conducted in accordance with the Declaration of Helsinki.

### Statistical analysis

Progression-free survival (PFS) was calculated from the first day of treatment until the date of radiological progression according to RECIST ver. 1.1., the date of death, or the last day of follow-up. Patient characteristics were compared according to representative gene alterations using the chi-squared test for categorical variables and the Mann–Whitney U test for continuous variables. The chi-squared test was also used to evaluate the relation between gene alterations and the response to immunotherapy for HCC. To compare PFS between groups, cumulative survival was calculated using the Kaplan–Meier method and compared using the log-rank test. *p* < 0.05 was considered statistically significant. All statistical analyses were performed using SPSS statistical software (version 21.0; SPSS, Chicago, IL, USA).

## Results

### Patient characteristics and samples for CGP

The data collection cut-off date was December 31, 2023. We retrospectively reviewed the list of the patients who received CGP tests at the three tertiary hospitals in Japan between February 2022 and November 2023. Of them, we identified the 52 patients with advanced HCC and collected data of them from medical records. Three patients (6%) for whom appropriate tissue samples were not available were evaluated using Foundation One® CDx Liquid, and 41 patients (79%) and 8 patients (15%) were evaluated using Foundation One® CDx and NCC OncoPanel, respectively (Table 1). Thirty-two patients (62%) underwent CGP after discontinuation or at the expected discontinuation of first-line treatment, 13 patients (25%) underwent CGP after discontinuation or at the expected discontinuation of second-line treatment, and seven patients (13%) underwent CGP after discontinuation or at the expected discontinuation of third- or later-line treatment. The median duration from tissue collection to CGP was 156 (range; 20–1044) days. The duration was 67 days for biopsy samples and 383 days for resected samples.

**Table 1:** Patient characteristics

	Total (*n* = 52)
Age, years	
Median	72.5
Range	55–84
Sex, *n* (%)	
Male	48 (92)
Female	4 (8)
Hepatitis B virus surface antigen, *n* (%)	
Positive	8 (15)
Negative	44 (85)
Hepatitis C virus antibody, *n* (%)	
Positive	14 (27)
Negative	38 (73)
Barcelona clinic liver cancer stage at the time of sample collection, *n* (%)	
A	4 (8)
B	26 (50)
C	22 (42)
Barcelona clinic liver cancer stage at the time of CGP test, *n* (%)	
B	25 (48)
C	27 (52)
Sample for comprehensive genome profiling test, *n* (%)	
Liver biopsy specimen	31 (60)
Previously resected tissue	18 (35)
Blood	3 (6)
Comprehensive genome profiling test, *n* (%)	
FoundationOne^®^ CDx	41 (79)
OncoGuide^™^ NCC OncoPanel system	8 (15)
Foundation One^®^ CDx liquid	3 (6)
Timing of comprehensive genome profiling test	
After discontinuation or at expected discontinuation of first-line treatment	32 (62)
After discontinuation or at expected discontinuation of second-line treatment	13 (25)
After discontinuation or at expected discontinuation of third or later-line treatment	7 (13)
Duration from tissue collection to CGP testing, day	
Median	156
Range	20–1044
Duration from CGP testing to molecular tumor board, day	
Median	27
Range	20–44

### Genomic landscape and tumor mutational burden

**Fig. 1:**
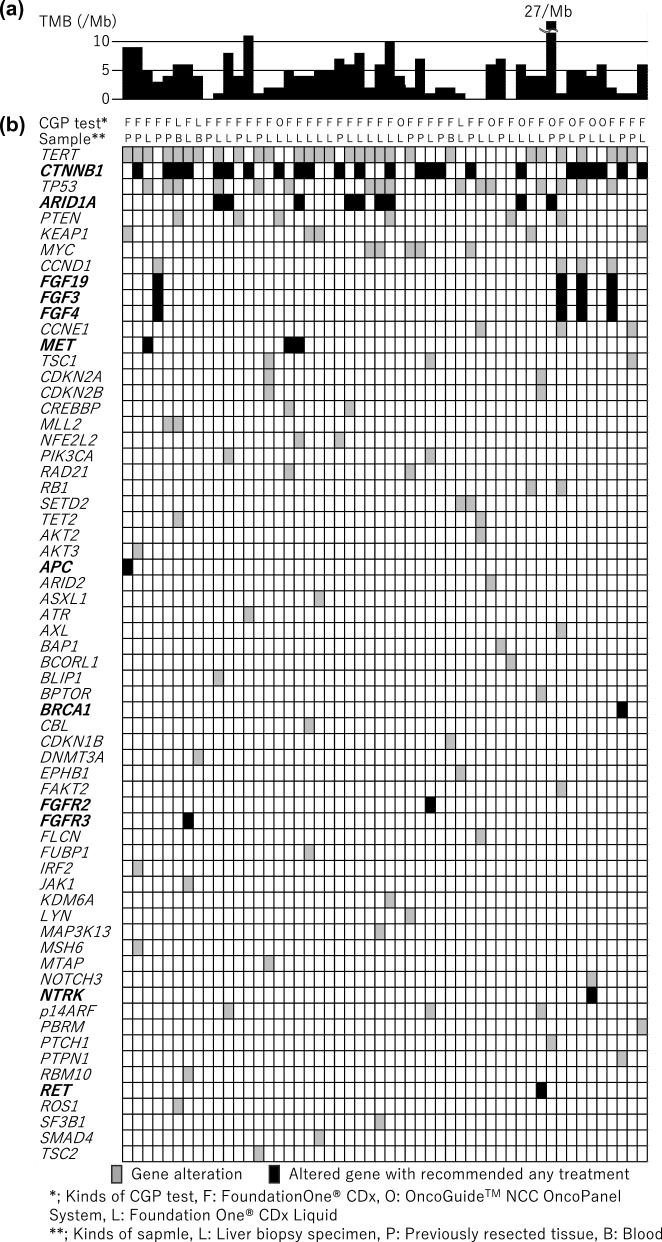
**a** Tumor mutational burden and **b** potentially actionable gene alterations in patients with advanced hepatocellular carcinoma evaluated by comprehensive genome profiling tests. Black boxes indicate gene alterations with recommendation of any treatments

Potentially actionable gene alterations are shown in Fig. 1. The mean and median number of somatic mutations per megabase (Mb) (i.e., the tumor mutational burden [TMB]) in the study population were 4.7 and 4 mutations/Mb, respectively. A high TMB, defined as 10 or more mutations/Mb, was observed in three patients (6%). One of these patients had 27.1 mutations/Mb, while the others had 10 and 11 mutations/Mb, respectively. No microsatellite instability-high or mismatch repair-deficient cases were observed. The most frequently observed variants were *TERT* promoter -124C > T in 29 patients (58%), followed by *CTNNB1* in 24 patients (45%), *TP53* in 16 patients (32%), *ARID1A* in 9 patients (18%), and *MYC* in 5 patients (10%).

### Treatment recommendations and selection after CGP

Among the actionable gene alterations, a molecular tumor board identified druggable gene alterations and determined the recommended treatment for each patient. Forty-five patients (87%) had one or more druggable gene alterations, and 34 patients (65%) were recommended any treatments based on gene alterations by the molecular tumor board (black boxes in Fig. 1). Treatments were covered by health insurance in 13 cases (25%) (Table 2).

**Table 2:** Gene alterations and treatment course in patients who were recommended any drugs under health insurance

Age	Sex	Gene panel	Gene	Alteration type	Recommended treatment	Actual treatment	Response to actual treatment
74	M	Foundation One^®^ CDx	*MET*	Amplification	Cabozantinib	Cabozantinib	Partial response
69	M	Foundation One^®^ CDx	*FGF19*	Amplification	Lenvatinib	Lenvatinib	Stable disease
75	M	Foundation One^®^ CDx	TMB	TMB 11/Mb	Pembrolizumab	Lenvatinib	Stable disease
73	M	Foundation One^®^ CDx	*MET*	Amplification	Cabozantinib	Best supportive care	No subsequent treatment
67	M	Foundation One^®^ CDx	*MET*	Exon 14 skipping	Cabozantinib	Cabozantinib	Stable disease
78	M	Foundation One^®^ CDx	TMB	TMB 10/Mb	Pembrolizumab	Best supportive care	No subsequent treatment
83	F	Foundation One^®^ CDx	*FGFR2*	Missense	Lenvatinib	Continuation of immunotherapy because of no tumor progression	
65	M	Foundation One^®^ CDx	*RET*	Amplification	Cabozantinib	Continuation of immunotherapy because of no tumor progression	
75	M	OncoGuide^™^ NCC Oncopanel	TMB	TMB 27.1/Mb	Pembrolizumab	Pembrolizumab	Stable disease
80	M	Foundation One® CDx	*FGF19*	Amplification	Lenvatinib	Continuation of immunotherapy because of no tumor progression	
73	M	Foundation One^®^ CDx	*FGF19*	Amplification	Lenvatinib	Continuation of immunotherapy because of no tumor progression	
71	M	OncoGuide^™^ NCC Oncopanel	*NTRK1* */TPM3*	Fusion	Larotrectinib	Larotrectinib	Partial response
76	M	Foundation One^®^ CDx	*FGF19*	Amplification	Lenvatinib	Continuation of immunotherapy because of no tumor progression	

For two patients with cytoplasmic mesenchymal–epithelial transition factor (*c-MET*) amplifications and one patient with a *MET* exon 14 skipping mutation, the molecular tumor board recommended cabozantinib. According to this recommendation, two patients received cabozantinib as second-line treatment after atezolizumab and bevacizumab combination therapy. One patient exhibited a good response to cabozantinib [14]; however, another patient discontinued cabozantinib due to high-grade proteinuria. Lenvatinib was recommended to four patients with *FGF19* amplification; this agent has a reported anti-tumor effect in *FGF19*-driven HCC [15]. In one patient who received lenvatinib, a long-term treatment response was achieved, although the dose was reduced due to thrombocytopenia. Lenvatinib was also recommended for a patient with *FGFR2* point mutation. Of the three patients with a high TMB, one received pembrolizumab and the remaining two did not, based on the lack of response to previous treatment with atezolizumab and bevacizumab. Finally, a patient with the *TPM3–NTRK1* fusion selected larotrectinib, a TRK inhibitor, as second-line treatment following the recommendation of the molecular tumor board and showed a good response; however, treatment was discontinued due to an infection and hepatic reserve deterioration. In total, five patients (10%) received the recommended treatment based on gene alterations as determined by the molecular tumor board. And other five patients underwent CGP test while receiving immunotherapy, and continued their immunotherapy after CGP test results were available, because their tumor progression was not confirmed by the data cut-off (Table 2). The recommended treatment based on gene alterations would be considered as the first choice for them if tumor progression was confirmed.

### Relationship between frequently observed gene alterations and patient characteristics or the efficacy of immunotherapy

We did not detect significant correlations between alterations in *hTERT*, *CTNNB1*, *TP53*, *ARID1A,* and *MYC* and clinicopathological findings, except for that patients with *TERT* alterations showed a lower frequency of HBs antigen positivity and higher rate of heavy alcohol consumption than did patients with wild-type *TERT* and patients with *ARID1A* mutations had a poor liver functional reserve (Table 3). Most patients selected atezolizumab and bevacizumab combination therapy as first-line treatment for HCC, and three patients received durvalumab and tremelimumab combination therapy. There were no differences in the response to treatment and PFS according to the *hTERT*, *CTNNB1*, *TP53*, *ARID1A*, and *MYC* mutation status (Table 4).

**Table 3:** Correlations between frequently observed gene alterations and patient characteristics

	*TERT*		*CTNNB1*		*TP53*	
	Presence (*n* = 29)	Absence (*n* = 23)	Presence (*n* = 24)	Absence (*n* = 28)	Presence (*n* = 16)	Absence (*n* = 36)
Age (median (range)), years old	73 (58–84)	72 (55–83)	72 (58–83)	73 (55–84)	73.5 (58–80)	72 (55–84)
Sex (male/female), *n*	28/1	20/3	23/1	25/3	15/1	33/3
Hepatitis B virus surface antigen (positive/negative), *n*	1/28	7/16	3/21	5/23	3/13	5/31
Hepatitis C virus antibody (positive/negative), *n*	9/20	5/18	6/18	8/20	5/11	9/27
Alcohol consumption (heavy/mild or none), *n*	18/11	7/16	14/10	11/17	8/8	17/19
MAFLD (presence/absence), *n*	3/26	4/19	2/22	5/23	1/15	6/30
Child–Pugh score (5/6/7), *n*	13/10/6	13/8/2	14/9/1	12/9/7	10/3/3	16/15/5
Vascular invasion (presence/absence), *n*	4/25	2/21	3/21	3/25	0/16	6/30
Extrahepatic lesion (presence/absence), *n*	12/17	11/12	11/13	12/16	8/8	15/21
α-fetoprotein (median (range)), ng/mL	12 (2–13,036)	7 (2–464,553)	12.5 (3–13,036)	8.5 (2–464,553)	10.5 (2–464,553)	10 (2–16,324)

	*ARID1A*		*MYC*		Tumor mutation burden	
	Presence(*n* = 9)	Absence(*n* = 43)	Presence(*n* = 5)	Presence(*n* = 47)	> 4 (*n* = 24)	≤ 4 (*n* = 28)
Age (median (range)), years	74 (58–84)	72 (55–84)	72 (60–76)	73 (55–84)	73 (60–84)	72 (55–84)
Sex (male/female), *n*	8/1	40/3	5/0	43/4	23/1	25/3
Hepatitis B virus surface antigen (positive/negative), *n*	0/9	8/35	1/4	7/40	4/20	4/24
Hepatitis C virus antibody (positive/negative), *n*	2/7	12/31	2/3	12/35	6/18	8/20
Alcohol consumption (heavy/mild or none), *n*	5/4	20/23	3/2	22/25	11/13	14/14
MAFLD (presence/absence), *n*	2/7	5/38	0/5	7/40	4/20	3/25
Child–Pugh score (5/6/7), *n*	3/2/4	23/16/4	1/2/2	25/16/6	10/9/5	16/9/3
Vascular invasion (presence/absence), *n*	0/9	6/37	1/4	5/42	1/23	5/23
Extrahepatic lesion (presence/absence), *n*	4/5	19/24	4/1	19/28	11/13	12/16
α-fetoprotein (median (range)), ng/mL	13 (7–770)	9 (2–464,553)	8 (4–1786)	11 (2–464,553)	12.5 (3–464,553)	8.5 (2–10,918)

**Table 4:** Correlations between frequently observed gene alterations and the response to immunotherapy

	*TERT*		*CTNNB1*		*TP53*	
	Presence (*n* = 29)	Absence (*n* = 23)	Presence (*n* = 24)	Absence (*n* = 28)	Presence (*n* = 16)	Absence (*n* = 36)
Objective response rate, %	31.0	34.8	37.5	28.6	37.5	30.6
Tumor control rate, %	93.1	82.6	79.2	96.4	87.5	88.9
Median progression-free survival, months	9.8	7.0	10.3	5.6	10.8	7.5

	*ARID1A*		*MYC*		Tumor mutation burden	
	Presence(*n* = 9)	Absence(*n* = 43)	Presence(*n* = 5)	Absence(*n* = 47)	> 4 (*n* = 24)	≤ 4 (*n* = 28)
Objective response rate, %	33.3	32.6	60.0	29.8	29.2	35.7
Tumor control rate, %	88.9	88.4	80.0	89.4	87.5	89.3
Median progression-free survival, months	9.9	7.8	15.3	7.5	7.8	8.9

## Discussion

In this study, we reviewed the results of CGP tests performed in clinical practice for patients with advanced HCC. Our CGP results were consistent with the previous results for the genetic landscape of HCC [16]; furthermore, our analysis of the impact of CGP results obtained under real-world conditions on treatment selection has important clinical significance for certain patients.

CGP tests were initially introduced in clinical practice for patients with various solid tumors at an advanced stage [17], providing a basis for determining alternative treatments or participating in clinical trials after the standard of care based on genetic alterations. In our cohort, one patient harbored the *TPM3–NTRK1* fusion gene, which is found at a relatively high frequency in thyroid or central nervous system tumors and is extremely rare in HCC [18]. The patient received a TRK inhibitor as second-line treatment under health insurance coverage based on CGP results and showed a good response. In addition, one of three patients with high TMB selected pembrolizumab. Although pembrolizumab has been shown to be effective in patients with high TMB, there is no evidence for the efficacy of this anti-programmed death receptor-1 antibody, even after progression to treatment using atezolizumab, an anti-programmed cell death ligand-1 antibody. We did not select pembrolizumab for the remaining two patients with tumor progression shortly after 6 weeks of atezolizumab plus bevacizumab therapy considering the relatively low TMB. Although pembrolizumab controlled tumor progression in a patient with 27.1 mutations/Mb, further studies are needed to determine which patients with high TMB are likely to benefit from pembrolizumab. Twenty-nine patients with alterations in several genes, including *CTNNB1*, *MET*, *PIK3CA*, *FGFR2*, and *BRCA1,* were potential candidates for clinical trials. We provided information about the clinical trials. However, no patients were enrolled in a clinical trial, although some visited sites of clinical trials. In a recent study in France, treatment selection for 15 patients with HCC and hepato-cholangiocarcinoma was based on the results of genomic testing, and some patients had a good response to treatment after progression under atezolizumab and bevacizumab combination therapy [19]. Health insurance systems and access to medicine differ substantially among countries. Eligibility for clinical trials is also updated occasionally and varies by region. Therefore, the impact of CGP on treatment selection may be constantly changing [20].

Of particular note, our results indicated that CGP tests can provide useful information for selecting second-line treatments after immunotherapy. For advanced HCC, several treatment options have been developed in the past decade, including first-line treatments as well as effective second- and later-line treatments, which are essential for improving prognosis in advanced HCC [21]. Effective second-line treatments are important, because not all patients can receive third- or later-line treatments due to tumor progression, deterioration of liver functional reserve, irreversible adverse events, or worsening of general condition [22]. However, data on the relative efficacies of second- or later-line treatments after immunotherapy are lacking. Furthermore, we do not have a clear picture of the role of CGP tests in clinical decision-making. For at least 12 patients (23%) in this cohort, accessible treatments covered by health insurance were recommended by the molecular tumor board, and those who received the recommended treatment exhibited a clinical benefit. We believed that these results support the clinical significance of CGP tests for the selection of second-line therapy after immunotherapy in patients with HCC, potentially benefiting a large number of patients.

The timing of CGP is a point of contention and depends on the purpose of the test. We performed CGP after the discontinuation of later-line treatment to provide a basis for the identification of alternative treatments based on gene alterations. For several patients, the information was useful for selecting second-line treatments; therefore, we provided information about CGP tests after the discontinuation or at the expected discontinuation of first-line treatment and conducted testing if the patients agreed. The most frequently observed druggable alterations in this cohort were *CTNNB1* mutations, for which β-catenin inhibitors are being evaluated in clinical trials (i.e., https://jrct.niph.go.jp/latest-detail/jRCT2080224780). Unfortunately, the study participants were not able to participate in clinical trials; in particular, 10 of 29 patients with *CTNNB1* mutations had already received second- or later-line treatment, which was an exclusion criterion. Earlier CGP tests may provide expanded treatment opportunities for patients and facilitate the development of new drugs for HCC. Some studies have identified predictive factors for the response to immunotherapy in HCC. For example, *CTNNB1* alterations are associated with a suppressive microenvironment and patients with *CTNNB1* alterations have a poorer response to immunotherapy than that of patients with wild-type *CTNNB1* [23, 24]. However, the reproducibility of these findings is unclear, and Montironi et al. reported that one of the two types of *CTNNB1* mutation positive tumors are considered to be responsive to immunotherapy [25]. Therefore, there is not sufficient evidence to justify CGP testing before first-line treatment.

In biliary tract cancer or other solid cancers, the development of precision medicine for second-line treatment resulted in increased drug availability, and CGP tests are positioned to become standard as companion diagnostic assay [26]. No agent based on gene alterations is available for HCC, and HCC has not yet reached that area. Even if druggable gene alterations can be detected, the recommended agents do not always produce favorable results due to deteriorated liver function and complications of cirrhosis, and therefore, the availability of agents for advanced HCC is currently limited [27]. We evaluated the effectiveness of the treatments recommended by the molecular tumor board using response to treatment, because this study included only patients who underwent CGP and the single-arm nature requires caution in interpreting time-to-event results such as PFS and OS. Moreover, because of the variety of treatment lines of the included patients, we selected anti-tumor effect as efficacy endpoint to directly assess the efficacy of treatment. However, we believed that the results of this study, coupled with successes in studies of other solid cancers and an increasing understanding of the molecular basis of HCC, suggest that treatment strategies based on gene alterations will be useful in the future.

CGP testing for patients without access to tumor tissue is one of the issues regardless of cancer [28]. Various factors including the kinds of sample, quality of sample, tumor cell content rate, intra-tumor and inter-tumor heterogeneity, and treatment can affect the sensitivity of detection of gene alterations [29]. It is important to note that TMB assessed via liquid biopsy can be also influenced by as the quantity of circulating tumor DNA (ctDNA) present in the blood or the potential interference from clonal hematopoiesis. When ctDNA levels are low, particularly in early or intermediate-stage cancers or in patients with minimal residual disease, the TMB estimation may be less reliable. Additionally, clonal hematopoiesis, which involves age-related mutations in hematopoietic stem cells, can introduce mutations unrelated to the tumor, potentially affecting the interpretation of TMB in liquid biopsy samples. In cases where tissue biopsy is available, TMB is typically considered more reliable due to the higher quantity and quality of tumor DNA, allowing for a more comprehensive assessment of the mutational burden. Therefore, as mentioned above, we prefer to collect tissue samples as much as possible or to use previously resected tissues. However, liquid biopsy has the advantages of easy access, non-invasiveness, and avoidance of biopsy complications, and with the rapid development of ctDNA technology [30], liquid biopsy remains a valuable, albeit potentially less precise, alternative for patients where only liquid biopsy is feasible.

The present study had several limitations, including a relatively small sample size restricted to the Japanese population, selection bias, and a short observation period. Therefore, further investigations and eventually large-scale studies are needed to definitely conclude the usefulness of CGP tests and precision medicine based on gene alterations for improving outcomes in patients with advanced HCC.

In conclusion, we reviewed the genomic profiles of patients with advanced HCC and subsequent treatment selection in clinical practice. A subset of patients with advanced HCC had druggable gene alterations, providing useful information not only for proposing alternative treatments but also for selecting more promising targeted treatments, especially second-line treatments after immunotherapy.

## Data Availability

The data support the findings of this study are available on request from the corresponding author, T.T. The data are not publicly available due to their containing information that could compromise the privacy of research participants.

## Abbreviations

HCC: Hepatocellular carcinoma
CGP: Comprehensive genomic profiling
CT: Computed tomography
MRI: Magnetic resonance imaging
RECIST: Response evaluation criteria in solid tumors
PFS: Progression-free survival
